# New data on limno-terrestrial rotifers of the world

**DOI:** 10.3897/BDJ.13.e171929

**Published:** 2025-12-30

**Authors:** Dzmitry Lukashanets, Nataliia Iakovenko, Diego Fontaneto, Miloslav Devetter, Karel Janko, Jolanta Ejsmont-Karabin, Irena Bielańska-Grajner, Bernard Hallet, Jerzy Smykla, Iryna Kozeretska, Vladlen Trokhymets, Vítězslav Plášek, Oleksii Redchenko, Murat Kaya, Yasin Hazer, Mariusz Wierzgoń

**Affiliations:** 1 Klaipeda University, Klaipeda, Lithuania Klaipeda University Klaipeda Lithuania; 2 Czech University of Life Sciences Prague, Prague, Czech Republic Czech University of Life Sciences Prague Prague Czech Republic; 3 Institute of Animal Physiology and Genetics AS ČR, Libechov, Czech Republic Institute of Animal Physiology and Genetics AS ČR Libechov Czech Republic; 4 University of Ostrava, Ostrava, Czech Republic University of Ostrava Ostrava Czech Republic; 5 Schmalhausen Institute of Zoology of NASU, Kyiv, Ukraine Schmalhausen Institute of Zoology of NASU Kyiv Ukraine; 6 National Research Council of Italy, Water Research Institute (CNR-IRSA), Verbania Pallanza, Italy National Research Council of Italy, Water Research Institute (CNR-IRSA) Verbania Pallanza Italy; 7 Biology Centre, Institute of Soil Biology AS ČR, České Budějovice, Czech Republic Biology Centre, Institute of Soil Biology AS ČR České Budějovice Czech Republic; 8 Nencki Institute of Experimental Biology, Polish Academy of Sciences, Warsaw, Poland Nencki Institute of Experimental Biology, Polish Academy of Sciences Warsaw Poland; 9 University of Silesia in Katowice, Katowice, Poland University of Silesia in Katowice Katowice Poland; 10 Catholic University of Louvain, Louvain-la-Neuve, Belgium Catholic University of Louvain Louvain-la-Neuve Belgium; 11 Institute of Nature Conservation, Polish Academy of Sciences, Krakow, Poland Institute of Nature Conservation, Polish Academy of Sciences Krakow Poland; 12 National Antarctic Scientific Center, Kyiv, Ukraine National Antarctic Scientific Center Kyiv Ukraine; 13 Taras Shevchenko National University of Kyiv, Kyiv, Ukraine Taras Shevchenko National University of Kyiv Kyiv Ukraine; 14 University of Opole, Institute of Biology, Opole, Poland University of Opole, Institute of Biology Opole Poland; 15 Institute of Molecular Genetics CAS, Prague, Czech Republic Institute of Molecular Genetics CAS Prague Czech Republic; 16 Istanbul Technical University, Istanbul, Turkiye Istanbul Technical University Istanbul Turkiye; 17 Bülent Ecevit University, Zonguldak, Turkiye Bülent Ecevit University Zonguldak Turkiye

**Keywords:** Bdelloidea, Rotifera, biogeography, bryophytes, lichens, soil, occurrence

## Abstract

**Background:**

Limno-terrestrial rotifers, particularly those of the order Bdelloidea, inhabit bryophytes, lichens, soils and other periodically moist terrestrial habitats. Despite their high abundance in all latitudinal zones, these rotifers remain poorly documented in biodiversity databases due to difficulties in preservation and morphological identification. As a result, the current knowledge of their global distribution is still highly fragmented and geographically biased, with the majority of species records concentrated in Europe (where the experts mostly collected material). Many regions like north-eastern Asia, North and South America, Africa and Australia remain under-represented in current knowledge on distribution of limno-terrestrial rotifers. Recent studies suggested that Bdelloidea exhibit distinct biogeographical patterns and potentially high levels of cryptic diversity and endemism, challenging the traditional view of the omnipresence of all microscopic taxa. Comprehensive, georeferenced occurrence data are essential to advance our understanding of bdelloid biodiversity and distribution, yet such data are still scarce in global platforms like the Global Biodiversity Information Facility (GBIF).

**New information:**

The dataset provides new, georeferenced data on the occurrence of rotifer species inhabiting limno-terrestrial habitats worldwide. For the first time, occurrence records for 48 rotifer species (including nominal taxonomic ‘subspecies’) are published in GBIF.

In particular, the dataset significantly expands the information on the distribution ranges of the bdelloid rotifers (order Bdelloidea) in GBIF. We contributed 5,651 new occurrence records of bdelloids rotifers, increasing the number of records in GBIF by 47.5% (or by 61.6% when considering only previously georeferenced records).

Moreover, we add 394 new records to the faunas of 49 of 56 studied countries, in 17 of which limno-terrestrial rotifers were studied for the first time.

Additionally, for 19 countries, records of bdelloid rotifers are now available in GBIF for the first time.

## Introduction

Limno-terrestrial microfauna represents a distinct ecological group of microscopic invertebrates inhabiting soil, litter, lichens, bryophytes and other environments that regularly undergo alternating periods of drying and humidity. This group includes various animal taxa such as rotifers (Rotifera), tardigrades (Tardigrada), nematodes (Nematoda) and others.

Within limno-terrestrial rotifers, bdelloids (Rotifera, Bdelloidea) are one of the most abundant and widely distributed groups. These obligate parthenogenetic organisms can survive under extreme environmental conditions (complete freezing, desiccation etc.) by entering a state of cryptobiosis – a temporary pause of metabolism (Ricci and Caprioli 2005, Shmakova et al. 2021). They also possess remarkable capabilities for passive dispersal, transporting via wind, water and other vectors (Fontaneto 2019). As a result, bdelloids inhabit a broad range of limno-terrestrial habitats and are distributed globally (Ricci 1987, Fontaneto et al. 2006, Fontaneto et al. 2007, Fontaneto et al. 2008). In certain environments, particularly those lacking larger invertebrates, bdelloids dominate in microinvertebrate communities, reaching large densities and playing a key role in ecosystem processes (Zawierucha et al. 2021).

However, bdelloid rotifers remain a poorly-studied taxon in terms of both biology and distribution. These animals cannot be preserved using traditional fixative chemicals, they are difficult to identify morphologically and are often overlooked in studies of biodiversity, soil biology etc.

Our current knowledge of bdelloid fauna and distribution remains significantly biased in geographical aspect. Most of original descriptions of bdelloids originate from Central and Western Europe, where pioneer studies were conducted (Donner 1965). The majority of new European bdelloid species descriptions comes from Austria (Donner 1949,Donner 1950a, Donner 1950b, Donner 1952), former Czechoslovakia (Bartoš 1951, Bartoš 1959), Germany (Janson 1893, Wulfert 1942, Wulfert 1960), the Netherlands (De Koning 1947), Romania (Rudescu 1960), Sweden (Bērziņš 1982) and UK (Davis 1873, Murray 1905, Murray 1906a, Bryce 1915). Although there were surveys of other regions, including Africa (Murray 1908, Murray 1911e, Murray 1911f, Milne 1916, Koste 1996), Asia (Murray 1906b, Hauer 1938, Bartoš 1963, Sudzuki 1981, Koste and Zhuge 1996), North America (Murray 1911b, Harring and Myers 1922, Burger 1948, Bateman and Davis 1980), South America (Murray 1907, Murray 1913, Donner 1980, Koste and Böttger 1989), Australia, New Zealand and Pacific Islands (Murray 1911a, Murray 1911c, Murray 1911d, Haigh 1963, Haigh 1970, Ricci et al. 2003), Antarctica (Murray 1910, Dartnall and Hollowday 1985), the majority of the first descriptions still arises from Europe. In recent decades, research efforts have expanded into previously poorly-studied areas of Asia (Song and Min 2015, Song and Lee 2020, Zeng et al. 2020, Song and Lee 2022, Wang et al. 2023, Song and Lee 2024, Wang et al. 2025), including tropical regions (Fontaneto and Ricci 2004, Jaturapruek and Maiphae 2025, Pokpongmongkol et al. 2025) and North America (Örstan 2018, Örstan 2021). Extensive research efforts were also recently made in Central and Eastern Europe (Kutikova 2005, Devetter 2007, Devetter 2009, Devetter 2010, Bielańska-Grajner et al. 2011, Iakovenko and Ovander 2011, Bielańska-Grajner et al. 2013, Lukashanets 2018).

The most complete identification key for bdelloid species is still that of Donner (1965), published in German and lacking more than 40 taxa described since (Örstan and Plewka 2017). Several more recent keys (e.g. Ricci and Melone (2000), Kutikova (2005), Bielańska-Grajner et al. (2013), Iakovenko et al. (2013)) are limited either to certain taxonomical ranks and groups or to regions (Poland, Russian Federation).

Many countries still have not been studied for their bdelloid fauna and taxonomy. For example, in the Arctic, bdelloid checklists are known mainly from Svalbard (Kaya et al. 2010, Coulson et al. 2024) with very little data from Greenland, Iceland, Russia and USA (Alaska) (De Ridder 1972). The main bulk of material in numerous surveys of Antarctica comes from Maritime Antarctic and Victoria Land (Iakovenko et al. 2015, Fontaneto et al. 2015), whilst data for East Antarctica are scarce (Lukashanets et al. 2021, Lukashanets et al. 2022). In Europe, the best studied continent in relation to bdelloid distribution, mostly fragmentary knowledge comes from Baltic States (Cimdiņš 1979, Zarubov and Cimdiņš 1990) and Scandinavia (Bērziņš 1982, Bērziņš 1987). Although the works of Fontaneto et al. (2006)and Fontaneto et al. (2007) greatly expanded the knowledge on mainland Italy, bdelloid fauna of Balkan countries, France, Portugal, Spain and Mediterranean Islands remains unknown for the most part. Other continents, especially central and north-eastern parts of Asia along with Africa and South America, still require extensive taxonomic and faunistic work.

Existing online repositories do not yet provide a comprehensive snapshot of bdelloid distribution. For example, as of July 2025, the Global Biodiversity Information Facility (GBIF) contains 11,894 occurrence records of bdelloids, from both aquatic and terrestrial ecosystems. From them, 2,590 (22%) are identified only to family or genus level and other 2,857 (or 24%) are noted as ‘Bdelloidea sp.’. Some current identifications of bdelloids in GBIF (according to the provided photos) have taxa misidentified not only to species, but even to genus and family level (personal observations).

Research over the past 20 years revealed specific biogeographical patterns in bdelloids (Fontaneto et al. 2008, Velasco-Castrillón et al. 2014, Iakovenko et al. 2015), supporting the concept that microscopic animals do have biogeography (Martínez et al. 2025). Furthermore, it has been claimed that bdelloid diversity is significantly underestimated due to cryptic species and high regional endemism (Velasco-Castrillón et al. 2014, Iakovenko et al. 2015). The most recent published annotated checklist includes more than 460 species (Segers 2007), but the actual number is believed to be much higher (Fontaneto et al. 2010).

All this highlights the strong need to collect new data, especially from underexplored regions, to fill gaps in our understanding of limno-terrestrial rotifer diversity.

In this work, we provide a dataset of limno-terrestrial rotifers from diverse regions across the globe, including all continents, several islands and archipelagoes and both polar areas. For our survey, samples were collected from a wide range of non-aquatic, but periodically moistened habitats, such as bryophytes (mosses and liverworts), lichens, algal mats, soil, needle, leaf or mixed litter. All rotifers (not only bdelloids, although they made up the majority of records) were identified to the lowest possible taxonomic rank.

The dataset has been submitted to GBIF with complete accompanying metadata. This includes detailed information on the occurrence sites: habitat (the substrate from which rotifers were isolated), biotope (the environment where the substrate was collected), locality (country and continent), elevation and exact coordinates. For each species, the density in the sample is provided.

The primary goal of this paper is to document and publish all verified global records of limno-terrestrial rotifers in GBIF. These data will also support future research on the local, regional and global distribution patterns of limno-terrestrial rotifers and will enable more in-depth taxonomic studies of this still poorly-understood taxon.

## Project description

### Title

ROTISFERA 'Global patterns of microinvertebrate distribution: does diversity decrease poleward in rotifers (Rotifera, Bdelloidea)?’ NDICI-GEO-NEAR/2022/434-092-0068

### Personnel

Dzmitry Lukashanets, Nataliia Iakovenko

### Study area description

The project encompasses all major regions of the Earth.

### Design description

To address the project's aims, we used samples collected starting from 1996 until the present in limno-terrestrial habitats throughout the world. More than 2,000 samples were analysed using conventional methods (analysis of rotifer morphology using light microscopy) resulting in creation of dataset with 5,729 rotifer species occurrences, 98.5% of which belong to bdelloids. The data cover all continents, including both polar zones and ranging from 0 to 4,610 m of altitude.

The following steps include estimating diversity patterns within defined geographical areas (alpha- and gamma-diversity, rarefaction curves, asymptotic diversity estimates) and globally – revealing the shape and prominence of the latitudinal diversity gradient using a hexagonal model and generalised additive model (GAM).

In parallel, the diversity of limno-terrestrial rotifers was assessed using a molecular approach. We applied metabarcoding of the COI mitochondrial gene for rotifers; however, this was not conducted at a global scale, but rather limited to Europe (from the Mediterranean to the Arctic). The results obtained from both methodological approaches were compared.

### Funding

European Union

## Sampling methods

### Study extent

All samples used in the dataset were collected either within the framework of several research projects focused on the biodiversity and ecology of microfauna, or as a part of other field activities. The latter included fieldwork in remote and hard-to-reach areas (tropical rainforests, polar and alpine regions), incidentally in field trips, within national Antarctic expeditions, during landing at cruises of research vessels etc. The samples have been stored in the repositories of the dataset’s main creators, Nataliia Iakovenko, Diego Fontaneto and Dzmitry Lukashanets. For the short-term storage, we used gradual drying and stored samples at room temperature or at +8°C, while, for long-term preservation freezing at -20–25°C was used (samples from other than polar latitudes were first gradually dried at room temperature before freezing).

### Sampling description

For the analysis of limno-terrestrial rotifers, sampling was performed mainly in forest ecosystems (taiga, boreal and mixed forests, subtropical forests, tropical rainforests), flatlands, urban areas and agricultural landscapes. In polar areas, we conducted sampling in moss tundra, at post-glacial moraine, in Antarctic oases and nunataks.

Samples of soil and litter were collected using 3.8 cm metal corer or metal scoops. Lichens, bryophytes and other vegetation were picked from the ground, tree barks and stones using tweezers or simply by hands (Fig. 2). The size of the samples varied from a few cm^3^ to several dozen cm^3^, depending on the amount of material available for collection. All samples were placed in paper or plastic bags and properly labelled. All sampling sites were georeferenced.

### Quality control

Specialists in taxonomy of Bdelloidea, Nataliia Iakovenko, Diego Fontaneto and Dzmitry Lukashanets, carried out all species identification while consulting other specialists (Aydin Örstan, Claudia Ricci). Primarily first descriptions together with the existing identification keys and reviews on the taxonomy of bdelloids were used. Georeferenced data were checked by placing latitudes and longitudes on a map.

### Step description

Sample processing included the following steps:

(i) Microscopic animals were isolated from each sample. To do this, bryophytes, lichens, soil, litter, timber, fungi or pieces of vegetation were washed through a system of sieves, followed by flotation and centrifugation in a sugar solution (Freckman and Virginia 1993). For some samples, the method for extracting moss-dwelling rotifers described by Peters et al. (1993) was used. For other samples, the substrates were washed repeatedly in distilled water.

(ii) Rotifers were counted and sorted under a binocular microscope (Olympus SZ61, Olympus SZX10, NR.3 Nikon SMZ1000) at magnification ranging between 10x and 100x.

(iii) For species identification, live rotifer individuals were transferred to slides and examined using light microscopy (Nikon Eclipse Ts2R, NIB-100F inverted microscope, Olympus CX43) at magnifications ranging from 400x to 1000x. First descriptions of taxa and the existing identification keys were used: Bartoš (1951), Bartoš (1959), Donner (1965), Kutikova (2005). Body dimensions and proportions were measured following the protocol of Iakovenko et al. (2013) and Iakovenko et al. (2015).

(iv) Rotifer densities were equalised to 25 cm^3^ (measured as 25 ml standard sample of the dried substrate using a plastic tube). In case when the sample volume was smaller, it was measured and rotifer densities were counted per sample and then recalculated to 25 cm^3^. The standard sample volume was chosen empirically as a minimal volume to be considered as a feasible and adequate representation of rotifer biodiversity in a sample.

## Geographic coverage

### Description

**Figure 1.**
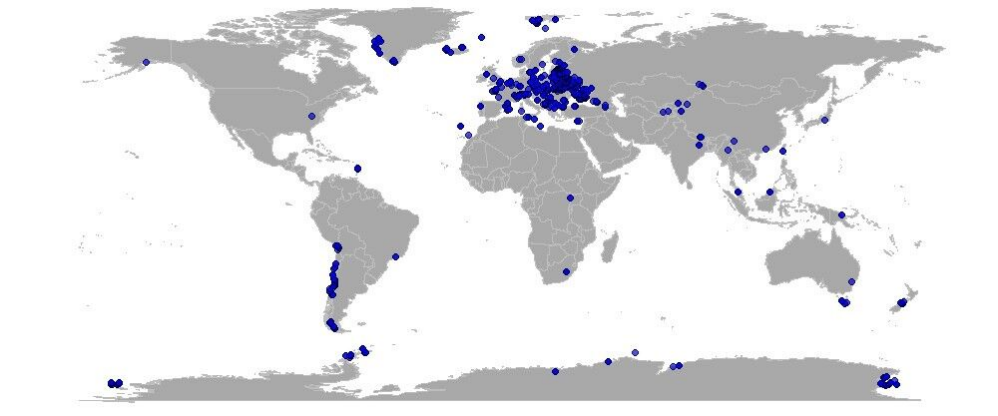
All sampling sites included in the dataset.

**Figure 2.**
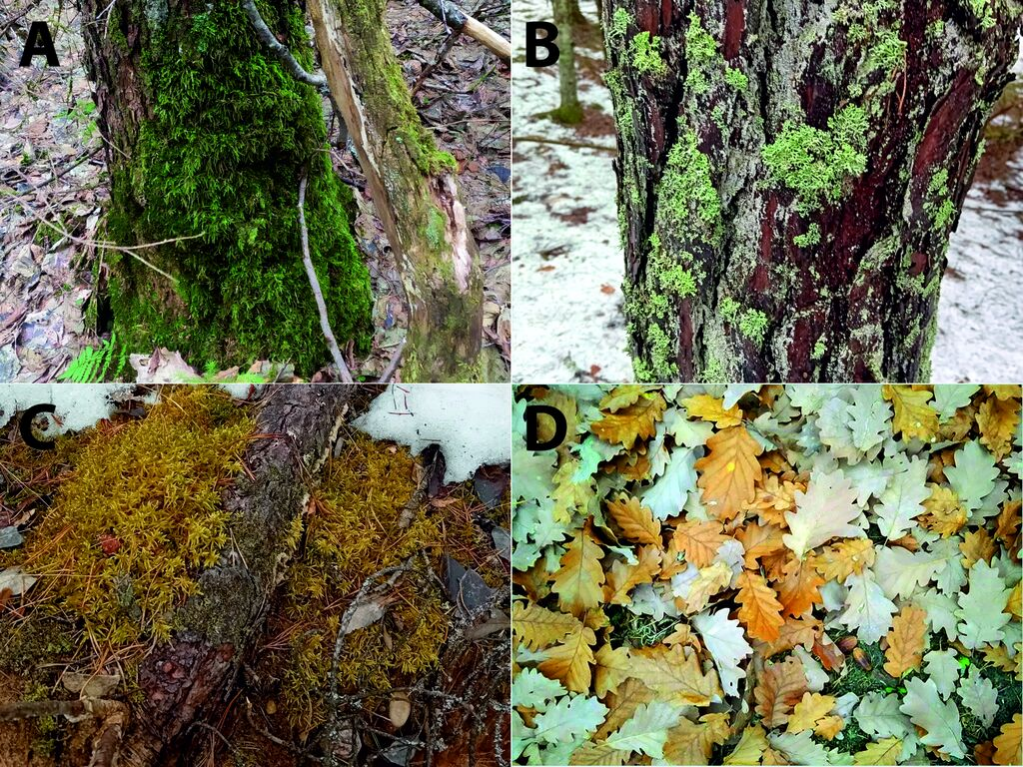
Examples of limno-terrestrial habitats: **A** moss on tree bark; **B** lichens on tree bark; **C** moss on ground; **D** leaf litter.

The dataset includes records collected throughout the world, spanning latitudes from 77.9° S to 78.2° N and covering all continents (Fig. 1):

(i) Europe – Austria, Belarus, Belgium, Bulgaria, Croatia, Czech Republic, Denmark, Estonia, Finland, France, Germany, Great Britain and Northern Ireland, Greece, Hungary, Italy, Latvia, Lithuania, Montenegro, Netherlands, North Macedonia, Norway, Poland, Portugal, Russian Federation (Caucasus and Karelia), Serbia, Slovakia, Spain, Sweden, Switzerland, Ukraine, also including the European Arctic (Iceland, Svalbard and Jan Mayen) and minor Mediterranean islands (Mallorca, Menorca, Malta, Pantelleria, Sardinia);

(ii) Asia – Armenia, Brunei, China, Cyprus, Georgia, India, Japan, Kyrgyzstan, Malaysia, Myanmar, Nepal, Russian Federation (southern Siberia), Taiwan, Tajikistan, Turkey;

(iii) Africa – Democratic Republic of Congo, Lesotho, Tunisia, also including Macaronesia (Madeira, Canary Islands);

(iv) North America – western Greenland, USA (Alaska, Maryland and Virginia), also including the Caribbean (Martinique);

(v) South America – Brazil, Chile;

(vi) Australia and Oceania – Australia (mainland and Tasmania), New Zealand, Papua New Guinea;

(vii) Antarctica – Maritime Antarctic (Antarctic Peninsula, Argentine Islands, James Ross Island, King George Island) and Continental Antarctica (Dronning Maud Land, Enderby Land, Victoria Land and Ross Sea Region).

## Taxonomic coverage

### Description

The dataset includes all records belonging to Phylum Rotifera, which we found in the samples of soil, bryophytes, lichens and other limno-terrestrial habitats. Monogonont rotifers (order Ploima) accounted for only 1.36% the records, whilst the majority of the records were bdelloids (order Bdelloidea). Species potentially new for science were indicated as *Genus* sp. or – in case of minor differences from the nominal description – as cf. It is noteworthy that 10 species amongst the ones that had been found were not typical for limno-terrestrial habitats, but rather occur in swamps and temporary water bodies (f. ex. *Philodina flaviceps*, *Synchaeta* sp.). Their encounter on terrestrial vegetation is most likely the result of the proximity to the waterbodies and/or high humidity of the habitat.

### Taxa included

**Table:** 

Rank	Scientific Name	Common Name
phylum	Rotifera	rotifers

## Temporal coverage

### Notes

1996–2025

## Usage licence

### Usage licence

Creative Commons Public Domain Waiver (CC-Zero)

## Data resources

### Data package title

New data on limno-terrestrial rotifers of the world

### Resource link

https://doi.org/10.15468/vymt96

### Alternative identifiers

https://cloud.gbif.org/eca/resource?r=limno-terrestrial_rotifera

### Number of data sets

1

### Data set 1.

#### Data set name

New data on limno-terrestrial rotifers of the world

#### Data format

Darwin Core Archive

#### Description

The dataset brings together all records of rotifers (Phylum Rotifera) found by N. Iakovenko, D. Lukashanets and D. Fontaneto worldwide in limno-terrestrial habitats – substrates that periodically undergo cycles of desiccation and hydration, but cannot be classified as temporary waterbodies, such as bryophytes, lichens, fungi, timber, upper layers of soil and litter (Lukashanets et al. 2025).

From 1996 to the present, we have collected and analysed more than 2,275 limno-terrestrial samples. Sampling covered all continents and over 50 countries, spanning from the Arctic to Antarctica, including most major biomes. The altitude of sampling sites ranged from 0 to 4,610 m above sea level. In total, the dataset includes 5,729 records of limno-terrestrial rotifers. We found 216 morphological species (including nominal ‘subspecies’ that, in bdelloids, are to be updated to species in future) belonging to 23 genera (nine of Monogononta and 14 of Bdelloidea). More than other 400 species-level entities were different in minor or major details from the original descriptions and potentially might be new taxa.

The submitted dataset is extensive and substantially contributes to the current knowledge of diversity and distribution of limno-terrestrial rotifers, which are still poorly known, especially outside Europe. We present it for the wider use in studies of microinvertebrate biodiversity and macroecology.

**Data set 1.** 

Column label	Column description
occurrenceID	An unique identifier for the record including the code of the country, year of sampling, ID of the sample and species name.
materialSampleID	An identifier of the sample, from which rotifers were isolated.
eventDate	Year of sampling.
decimalLatitude	Latitude in decimal degrees.
decimalLongitude	Longitude in decimal degrees.
verbatimElevation	Elevation above sea level in metres.
continent	The name of the continent where sampling occurred.
country	The name of the country where sampling occurred.
countryCode	ISO 3166-1 alpha-2 country code.
stateProvince	The name of the next smaller administrative region than country, where sampling occurred (used only for USA).
island	The name of the island where sampling occurred (if country includes different islands or mainland and islands).
islandGroup	The name of the archipelago where sampling occurred (if country includes different islands or mainland and islands).
locationRemarks	The name of biotope where sampling occurred.
habitat	A description of the type of the sample, from which rotifers were isolated.
basisOfRecord	The specific nature of the data record.
scientificName	The full scientific name, with authorship and date information.
individualCount	The number of individuals present at the time of the Occurrence.
identifiedBy	Name and surname of the person who identified the species.
identifiedByID	ORCID of the person who identified the species.
sampleSizeValue	A numeric value for a measurement of the size of the sample.
sampleSizeUnit	The unit of measurement of the size of the sample.
kingdom	The full scientific name of the kingdom in which the species is classified.
phylum	The full scientific name of the phylum in which the species is classified.
class	The full scientific name of the class in which the species is classified.
order	The full scientific name of the order in which the species is classified.
family	The full scientific name of the family in which the species is classified.
genus	The full scientific name of the genus in which the species is classified.
specificEpithet	The name of the species epithet of the scientificName.
infraspecificEpithet	The name of the infraspecies epithet of the scientificName.
taxon rank	The taxonomic rank of the record.
identificationQualifier	A controlled value to express the identifier's doubts about identification.
scientificNameAuthorship	The authorship information for the scientificName.
recordedBy	Name and surname of the personwho collected the sample, from which rotifers were isolated.

## Additional information

### Limno-terrestrial rotifers: overview of taxonomy

The dataset includes a total of more than 600 taxa identified to species and below species level. The majority of the records belong to order Bdelloidea (representing over 98% of all species). Within Bdelloidea, the most diverse families are Habrotrochidae (particularly the genus *Habrotrocha* with 264 species) and Philodinidae, notably the genera *Macrotrachela* (178), *Mniobia* (81) and *Philodina* (54 species). The genus *Adineta* (family Adinetidae) is represented with 43 species (Table 1).

The dataset additionally includes records from the order Ploima, comprising 19 species in total. Amongst these, the most represented genera are *Lecane* (Lecanidae), *Encentrum* (Dicranophoridae) and *Cephalodella* (Notommatidae), along with several other genera with 1–2 species each (Table 1).

Overall, the taxonomic scope of the dataset reflects a comprehensive sampling of limno-terrestrial rotifers, with a focus on bdelloid taxa, which are typically dominant in these habitats.

The number of species recorded in different types of habitat varied from 205 (mosses) to seven (fungi); however, some bias related to different number of samples collected per habitat type is possible (Table 2).

### New records

Our dataset contains 394 new records for 49 of 56 studied countries, in 17 of which limno-terrestrial rotifers were studied for the first time. These data are summarised in Table 3.

However, these new records include only species that are identified as corresponding to the existing descriptions. At the same time, more than 400 morphologically distinctive species in this database either had minor differences from these descriptions (designated as cf.) or exhibited new features (designated as Genus sp.) and, thus, might potentially be species new for science. The highest number of such entities were found in the least studied regions (Arctic, Antarctica, South America, Africa). This calls for increasing the efforts for not only faunistic surveys, but primarily for the thorough taxonomical work aiming at describing new species characteristic for such regions, combining both morphological and morphometrical analysis with DNA barcoding.

## Figures and Tables

**Table 1.** Taxonomic ranks of rotifers (with the number of species) recorded in limno-terrestrial habitats.

**Class**	**Order**	**Family**	**Genus**	**Number of species (including subspecies and species-level entities different from nominal description)**
Eurotatoria	Bdelloidea	Adinetidae	* Adineta *	43
			* Bradyscela *	2
		Habrotrochidae	* Habrotrocha *	264
			* Otostephanos *	11
			* Scepanotrocha *	11
		Philodinavidae	Philodinavus*	1
		Philodinidae	* Ceratotrocha *	6
			* Didymodactylos *	1
			* Dissotrocha *	2
			* Macrotrachela *	178
			* Mniobia *	81
			* Philodina *	54
			* Pleuretra *	12
			* Rotaria *	8
	Ploima	Dicranophoridae	* Encentrum *	4
			* Wierzejskiella *	1
		Lecanidae	* Lecane *	5
		Lepadellidae	Colurella*	1
			* Lepadella *	2
			* Squatinella *	1
		Lindiidae	Lindia*	1
		Notommatidae	* Cephalodella *	3
		Synchaetidae	Synchaeta*	1

* only species different from nominal descriptions were recorded.

**Table 2.** Number of recorded taxa (families, genera, species) per type of habitat.

**Habitat**	**Number of families**	**Number of genera**	**Number of species (including subspecies)**
Bryophytes (excluding Sphagnum mosses)	7	19	205
Lichens	6	13	80
Sphagnum mosses	9	17	40
Algae / algal mats	5	8	33
Fungi	2	3	7
Grass	3	6	31
Leaf litter	6	13	67
Needle litter	5	13	63
Mixed litter	4	9	60
Soil	6	14	76
Sand / stones	3	7	26

**Table 3.** Total and new records of limno-terrestrial rotifers in the database.

**Country/ Continent**	**Number of records**	**Number of species found (including subspecies and species designated as cf. or Genus sp.)**	**Species new for the country/continent**
Antarctica	850	44	Data published
Armenia*	4	3	All new for the country
Australia (mainland)	6	6	–
Australia (Tasmania)	12	6	*Habrotrocha angusticollis angusticollis*, *H. aspera*, *H. placida*
Austria	13	8	–
Belarus	236	58	Data published
Belgium	17	10	*Macrotrachela ehrenbergii*, *M. nana nana*, *M. plicata plicata*, *Mniobia russeola*, *Mn. tetraodon*, *Philodina plena*
Brazil	1	1	–
Brunei*	7	5	All new for the country
Bulgaria*	66	29	*Adineta barbata*, *Bradyscela clauda*, *B. granulosa*, *Habrotrocha flaviformis*, *H. gracilis gracilis*, *H. mediocris*, *H. pavida*, *H. puella puella*, *H. pusilla pusilla*, *Macrotrachela concinna*, *M. ehrenbergii*, *M. libera libera*, *M. multispinosa multispinosa*, *M. papillosa*, *M. plicata hirundinella*, *M. punctata*, *M. quadricornifera quadricornifera*, *M. quadricornifera quadricorniferoides*, *Mniobia bredensis*, *Mn. russeola*, *Mn. scabrosa*, *Philodina plena*, *Ph. vorax*, *Pleuretra brycei*, *Rotaria sordida sordida*
Chile	81	25	*Mniobia scabrosa*
China	19	12	Data published
Croatia	6	6	*Didymodactylos carnosus*, *Macrotrachela quadricorifera quadricornifera*, *M. timida timida*, *Pleuretra lineata*
Cyprus*	34	17	All new for the country
Czech Republic	107	45	–
Congo*	15	10	All new for the country
Denmark (mainland)*	7	7	All new for the country
Denmark (Greenland)	90	34	All new for the country
Estonia*	93	29	All new for the country
Finland	18	13	*Habrotrocha constricta*, *H. pavida*, *H. puella puella*, *Macrotrachela concinna*, *M. ehrenbergii*, *M. habita*, *M. multispinosa multispinosa*, *Mniobia russeola*, *Philodina plena*, *Rotaria sordida sordida*
France (mainland)	157	37	*Adineta steineri*, *A. vaga minor*, *Habrotrocha elegans*, *H. minuta*, *Macrotrachela nana nana*, *M. timida timida*, *Mniobia tentans*
France (Martinique)*	10	5	All new for the region
Georgia*	3	3	All new for the country
Germany	72	31	*Habrotrocha minima*, *H. novemdens*, *H. pulchra*, *H. pusilla pusilla*
Greece	2	2	*Macrotrachela timida timida*
Hungary	98	29	*Habrotrocha gracilis gracilis*, *H. pavida*, *H. puella puella*, *H. sylvestris*, *Mniobia edmondsoni*, *Philodina proterva*
Iceland	50	20	*Didymodactylos carnosus*, *Macrotrachela quadricornifera quadricorniferoides*, *M. quadricornifera scutellata*, *Mniobia magna*, *Mn. scarlatina*
India	2	2	–
Italy (mainland)	276	69	Data published
Italy (Mediterranean islands)	15	7	All new for the region
Japan	3	3	*Habrotrocha angusticollis reversa*, *Habrotrocha pusilla nuda*
Kyrgyzstan*	12	8	All new for the country
Latvia	94	34	*Adineta steineri*, *Ceratotrocha franzi*, *Didymodactylos carnosus*, *Habrotrocha bidens*, *H. elusa vegeta*, *H. fusca*, *H. fuscochlaena*, *H. gracilis gracilis*, *H. rosa*, *H. tridens*, *Macrotrachela aculeata*, *M. habita*, *M. libera*, *M. multispinosa multispinosa*, *M. papillosa*, *M. plicata hirundinella*, *M. quadricornifera quadricornifera*, *Mniobia magna*, *Mn. montium*, *Mn. russeola*, *Mn. scarlatina*, *Philodina plena*, *Ph. vorax*, *Rotaria sordida*, *Scepanotrocha rubra*
Lesotho*	50	13	All new for the country
Lithuania	11	9	*Adineta steineri*, *Rotaria sordida sordida*, *R. sordida fimbriata*
Malaysia	9	5	All new for the country
Malta*	3	3	All new for the country
Montenegro*	42	17	All new for the country
Myanmar	3	3	*Macrotrachela habita*
Nepal*	16	5	All new for the country
Netherlands	19	15	*Adineta steineri*, *H. gracilis gracilis*, *H. tripus*, *Macrotrachela quadricornifera scutellata*, *Mniobia tentans*, *Philodina intermedia*
New Zealand	38	11	*Ceratotrocha velata*, *Habrotrocha visa*, *Macrotrachela bilfingeri*, *M. multispinosa multispinosa*
North Macedonia*	24	13	All new for the country
Norway (mainland)*	11	9	All new for the country
Norway (Jan Mayen and Bear Island)*	19	13	All new for the region
Norway (Svalbard)	202	45	*Adineta elongata*, *Ceratotrocha velata*, *Habrotrocha pavida*, *H. pusilla pusilla*, *Macrotrachela bilfingeri*, *M. crucicornis*, *M. decora*, *M. induta*, *M. intermedia*, *M. oblita*, *M. timida*, *Mniobia scarlatina*
Papua New Guinea*	2	2	All new for the country
Poland	518	86	Data published
Portugal (mainland)*	11	5	*Pleuretra lineata*
Portugal (Madeira)	23	15	–
Russian Federation	16	9	–
Serbia*	47	20	All new for the country
Slovakia	7	7	*Habrotrocha pusilla pusilla*, *Mniobia tentans*
Spain (mainland)	2	2	–
Spain (Mediterranean islands)	23	14	–
Spain (Canary islands)	2	2	*Habrotrocha gracilis gracilis*
Sweden	60	33	*Habrotrocha flaviformis*, *H. minima*, *H. pavida*, *H. tripus*, *Macrotrachela insolita*, *M. plicata hirundinella*, *M. quadricornifera scutellata*, *Mniobia loxocorona*, *Scepanotrocha simplex*
Switzerland	16	12	*Habrotrocha minima*, *Macrotrachela libera libera*, *Pleuretra lineata*
Taiwan	2	1	–
Tajikistan*	7	5	All new for the country
Tunisia*	2	2	*Rotaria sordida sordida*
Turkey	77	32	*Habrotrocha flaviformis*, *H. pavida*, *H. puella puella*, *H. pulchra*, *H. serpens*, *Macrotrachela inermis*, *M. multispinosa multispinosa*
Ukraine	1930	139	*Habrotrocha humilis*
UK (England)	41	27	*Habrotrocha minima*, *H. puella puella*, *Macrotrachela inermis*, *M. libera*, *M. minuta minuta*
UK (Northern Ireland)	7	6	*Habrotrocha elegans*, *H. filum*
USA	14	11	–

* the country or region was studied for the first time.
